# Tubacin, an HDAC6 Selective Inhibitor, Reduces the Replication of the Japanese Encephalitis Virus via the Decrease of Viral RNA Synthesis

**DOI:** 10.3390/ijms18050954

**Published:** 2017-05-01

**Authors:** Chien-Yi Lu, Yi-Chih Chang, Chun-Hung Hua, Chieh Chuang, Su-Hua Huang, Szu-Hao Kung, Mann-Jen Hour, Cheng-Wen Lin

**Affiliations:** 1 Department of Medical Laboratory Science and Biotechnology, China Medical University, Taichung 40402, Taiwan; cylu0424@gmail.com (C.-Y.L.); yichih@mail.cmu.edu.tw (Y.-C.C.); alicechuang7816@gmail.com (C.C.); 2 Department of Otolaryngology, China Medical University Hospital, Taichung 40402, Taiwan; flowererix@yahoo.com.tw; 3 Department of Biotechnology, Asia University, Taichung 41354, Taiwan; shhuang@asia.edu.tw; 4 Department of Biotechnology and Laboratory Science in Medicine, National Yang Ming University, Taipei 11221, Taiwan; szkung@ym.edu.tw; 5 School of Pharmacy, China Medical University, Taichung 40402, Taiwan

**Keywords:** Japanese encephalitis virus, histone deacetylase 6, tubacin, heat shock protein 90 (Hsp90), non-structural protein 5 (NS5)

## Abstract

Japanese encephalitis virus (JEV), a neurotropic flavivirus, annually causes over 30,000 Japanese Encephalitis (JE) cases in East and Southeast Asia. Histone deacetylases (HDACs) modulate lysine acetylation of histones and non-histone proteins, regulating many processes including inflammation and antiviral immune response. This study investigated antiviral activity of pan- and selective-HDAC inhibitors as host-targeting agents against JEV. Among HDAC inhibitors, selective HDAC6 inhibitors (tubastatin-A (TBSA) and tubacin) concentration-dependently inhibited JEV-induced cytopathic effect and apoptosis, as well as reduced virus yield in human cerebellar medulloblastoma cells. The 50% inhibitory concentration (IC50) values of virus yield was 0.26 μM for tubacin and 1.75 μM for TBSA, respectively. Tubacin (IC50 of 1.52 μM), but not TBSA, meaningfully blocked the production of intracellular infectious virus particles. In time-of-addition assays, the greatest potency of antiviral activity was observed in the mode of pre-treatment with tubacin (IC50 of 1.89 μM) compared to simultaneous (IC50 of 4.88 μM) and post-treatment (IC50 of 2.05 μM) modes. Interestingly, tubacin induced the hyperacetylation of a HDAC6 substrate Hsp90 and reduced the interaction of Hsp90 with JEV NS5 protein. Novobiocin, an Hsp90 inhibitor, diminished the NS5 protein amount and virus replication in JEV-infected cells. Meantime, tubacin suppressed the NS5 expression and antisense RNA genome synthesis in infected cells. Tubacin-induced Hsp90 hyperacetylation was suggested to influence the NS5 activity in JEV replication. Therefore, tubacin had a high potential of a host-targeting agent against JEV, exhibiting preventive and therapeutic activities against JEV infection.

## 1. Introduction

Japanese encephalitis virus (JEV), a mosquito-borne flavivirus, causes Japanese Encephalitis (JE) with poliomyelitis-like paralysis, aseptic meningitis, and encephalitis. Over 30,000 JE cases, that included 10,000 deaths, are reported annually in East and Southeast Asia, along with northern Australia due to the humid climate and transmission vectors [1,2,3]. JEV antigens and genomes are identified in the thalamus, basal ganglia, brainstem, cerebellum, cerebral cortex, and spinal cord from JE patients [4,5]. JEV contains a single strain positive sense RNA genome with a single, long ORF that encodes a polyprotein divided into three structural proteins (capsid (C), membrane (prM/M), and envelope (E)) and seven non-structural proteins (NS1, NS2A, NS2B, NS3, NS4A, NS4B, and NS5) [6,7]. Viral NS2B-NS3 protease mediates the proteolytic processing of this large polyprotein [8]. NS5 consists of a multifunctional protein containing RNA-dependent RNA polymerase and methyltransferase that are involved in viral RNA replication [9]. Non-structural proteins, like NS3 and NS5, are involved in forming viral replication complexes along with host factors [10]. Like the hepatitis C virus (HCV), NS5A interacts with heat shock protein 90 (Hsp90) and the FK506-binding protein FKBP8 to form the viral replication complexes in the RNA replication [11].

Histone acetyltransferases (HATs) and histone deacetylases (HDACs) modulate acetylation of lysine residues in histones and non-histone proteins [12]. HATs transfer the acetyl group via acetyl-CoA and HDACs/SIRTs deacetylate ε-N-acetylated lysine residues using Zn^2+^ (HDACs) or NAD^+^ (SIRTs) as cofactors [13]. Class I HDACs (HDAC1, 2, 3, and 8) are ubiquitously expressed HDACs; classes IIa (HDAC4, 5, 7, and 9) and IIb (HDAC6, and 10) are limitedly expressed in certain cell types [14,15]. Among HDACs, HDACA6 is mainly in the cytoplasm. Acetylation of non-histone proteins alters the binding affinity of host proteins, such as chaperones (Hsp90 and cyclophilin A) to the client proteins, and is also involved in the regulation of protein stability and DNA binding activity, for example p53, p73, Smad7, and c-Myc [16]. Recently, HDAC6 has been demonstrated to remove the acetylation of retinoic acid-inducible gene-I (RIG-I) during acute RNA virus infection, and is involved in RIG-I-dependent innate antiviral immune response [17]. In addition, HDAC inhibitors exhibit therapeutic potential to viral infection [18,19,20,21,22,23]. Pan-HDAC inhibitors such trichostatin A (TSA), suberoylanilide hydroxamic acid (SAHA), and valproic acid (VPA) block the zinc-containing catalytic domain of HDACs [18,19]. TSA reduces the number of viral genomes in Herpes Simplex Virus-1 infected cells [20]. SAHA activates HIV-I from latency period [21]. Tubastatin-A (TBSA), an HDAC6-selective inhibitor, decreases in viral RNA concentration in hepatocyte cell infected with HCV replicon [22]. SIRT1 inhibitor sirtinol reduces the DNA replicative intermediate and 3.5-kb mRNA during hepatitis B virus (HBV) replication [23]. Therefore, modulating the acetylation of histones and non-histone proteins plays a crucial role in viral replication.

This study investigates the antiviral activity and related mechanisms of pan- and selective-HDAC inhibitors against JEV. Pan- (TSA and VPA) and selective-HDAC (TBSA and tubacin) inhibitors were initiated to their inhibitory action on JEV-induced cytopathic effect (CPE) and apoptosis in human cerebellar medulloblastoma TE671 cells. Antiviral activity of pan- and selective-HDAC inhibitors was evaluated by their inhibitory effect on JEV-induced cytopathic effect (CPE) and apoptosis, virus yields in cultured supernatants, and intracellular virus titers. The antiviral mechanism(s) of HDAC inhibitors were determined using attachment, time-of-addition, and viral RNA synthesis assays.

## 2. Results

### 2.1. Antiviral Activity of Pan- and Selective-HDAC Inhibitors Against JEV

**Figure 1:**
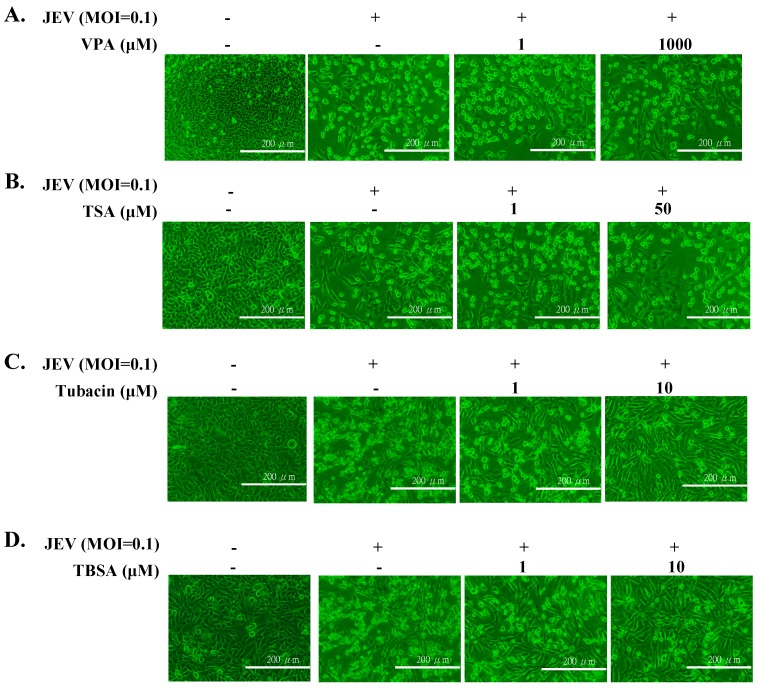
Reduction of Japanese encephalitis virus (JEV)-induced cytopathic effects by histone deacetylase (HDAC) inhibitors. TE671 cells were infected with JEV at an multiplicity of infection (MOI) of 0.1, and immediately treated with VPA (**A**), TSA (**B**), tubacin (**C**), and Tubastatin-A (TBSA) (**D**), respectively. Virus-induced cytopathic effect was photographed 36 h post treatment by light microscopy. Scale bar = 200 µm.

**Figure 2:**
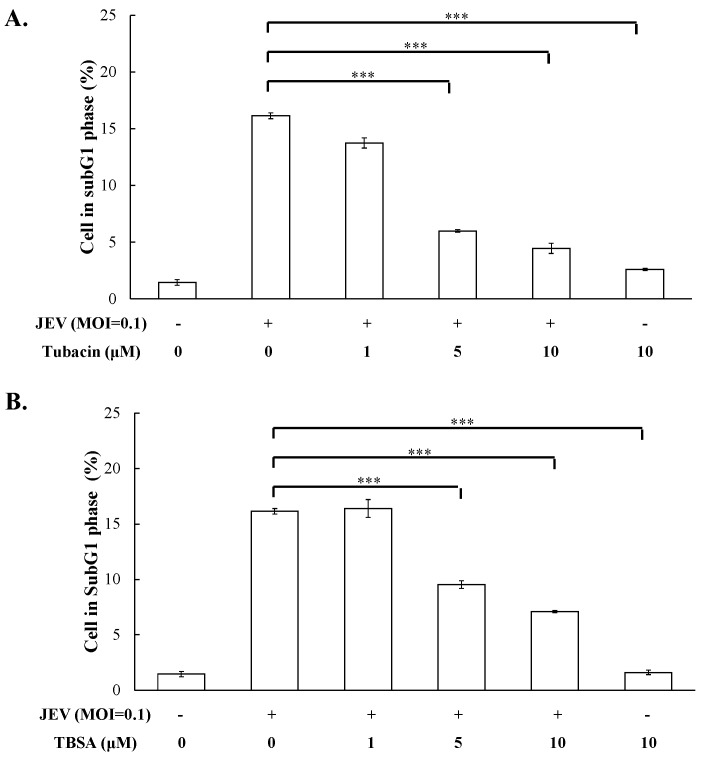
Inhibition of JEV-induced apoptosis by tubacin and TBSA. Cells were infected with JEV (MOI of 0.1), and immediately treated with tubacin (**A**) and TBSA (**B**). After 36 h incubation, cells were harvested, stained by PI dye, and then analyzed using flow cytometry. The percentage of sub-G1 phase in infected cells was presented. *** *p* value < 0.001 compared with mock-treated infected cells.

**Figure 3:**
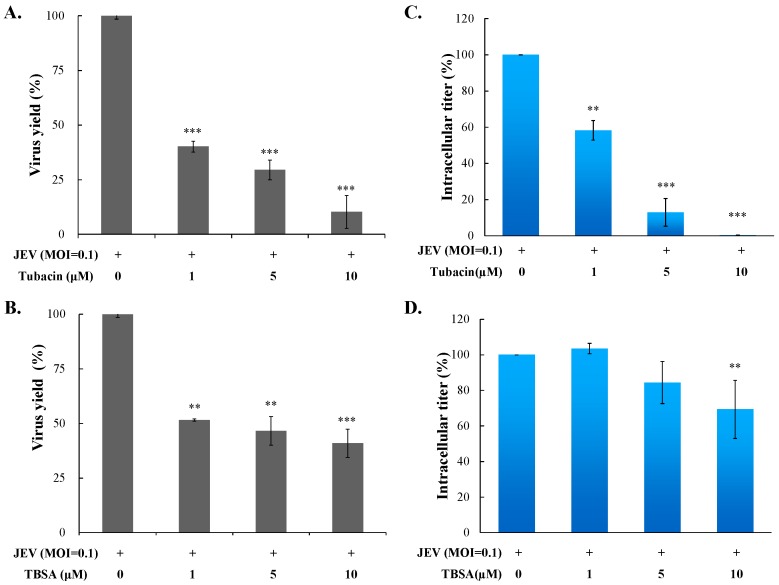
Suppression of virus yield and intracellular virion production by tubacin and TBSA. Cells were infected with JEV and immediately treated with indicated concentration of tubacin and TBSA. Virus yield in supernatant from infected cells treated with or without tubacin (**A**) and TBSA (**B**) was measured by plaque assay 36 h post infection. In intracellular virion production assay, the infected cells treated with or without tubacin (**C**) and TBSA (**D**) were lysed by three freeze-thaw cycles. The titer of intracellular infectious particles was determined by plaque assay. ** *p* value < 0.01; *** *p* value < 0.001 compared with untreated infected cells.

Cytotoxicity of TSA, VPA, TBSA, and tubacin was firstly evaluated using MTT assays (Figure S1A–D), then the optimal test concentration of these four HDAC inhibitors with less cytotoxicity was used for subsequent antiviral assays, including cytopathic effect inhibition, apoptosis reduction, virus yield, and intracellular infectious viral particle tests. In cytopathic effect inhibition assay, microscopic photography indicated tubacin and TBSA, but not VPA and TSA, concentration-dependently reduced cytopathic effect of JEV infection at an MOI of 0.1 36 h post infection (Figure 1). Meanwhile, apoptotic cell fraction of infected cells in the presence or absence of HDAC inhibitors was measured using propidium iodide flow cytometric assay (Figure 2). JEV infection at an MOI of 0.1 caused the appearance of a sub-G1 (apoptotic) fraction. Tubacin and TBSA significantly diminished cell apoptosis post infection (Figure 2A,B). The inhibitory activity of tubacin and TBSA on JEV-induced cytopathic effect (CPE) was associated with the reduction of apoptosis of infected cells in a concentration-dependent manner. Moreover, supernatant virus yield determined using the plaque assay demonstrated that tubacin and TBSA, markedly inhibited JEV production in human cerebellar medulloblastoma cells (Figure 3A,B). The 50% inhibitory concentration (IC50) values on virus yield were 0.26 μM for tubacin and 1.75 μM for TBSA, respectively. Notably, tubacin, but not TBSA, meaningfully blocked the intracellular production of infectious virus particles (Figure 3C,D). The IC50 value of tubacin on intracellular infectious virus titers was 1.52 μM. Among these HDAC inhibitors, tubacin showed the highest antiviral potency against JEV.

### 2.2. Preventive and Therapeutic Activities of Tubacin against JEV Infection

**Figure 4:**
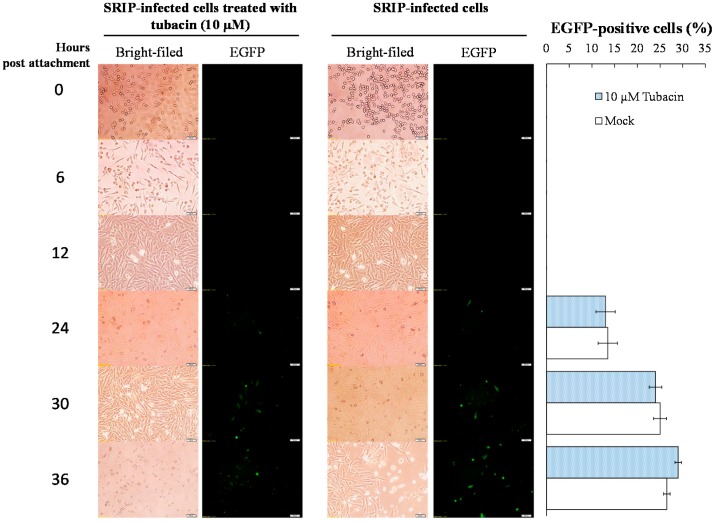
Real-time fluorescence imaging of the JEV SRIP-driven EGFP reporter for analyzing attachment inhibition by tubacin. Cells were infected with JEV SRIPs (10 TCID50), and then immediately treated with or without 10 µM tubacin for 1 h at 4 °C. After washing twice with PBS, bright-field and fluorescence images of infected cells were taken 0, 6, 12, 24, 30, and 36 h post infection (**left panel**). The percentage of EGFP-positive cells indicating SRIP replication in vitro was also calculated (**right panel**). Scale bar = 50 µm.

**Figure 5:**
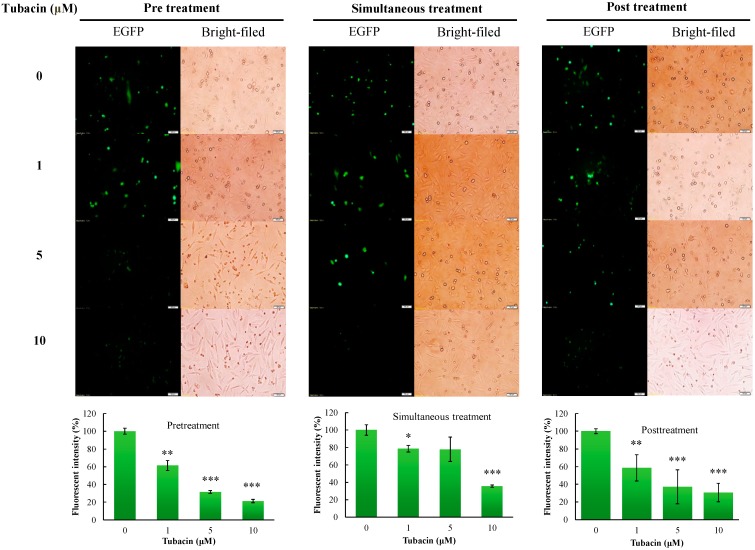
Time-of-addition assay for analyzing antiviral action of tubacin against JEV SRIPs. SRIP-infected cells were treated with tubacin 1 h prior (pre) (**left**), simultaneous (**middle**), or 1 h post (**right**) infection. Bright-field and fluorescence images of infected cells were taken 36 h post infection (**upper**). Green fluorescence intensity of SRIP-driven EGFP reporter in infected cells was quantified using Image J, and then relative intensity was normalized by the total of cells (**bottom**). * *p* value < 0.05; ** *p* value < 0.01; *** *p* value < 0.001 compared with untreated infected cells. Scale bar = 50 µm.

To ascertain antiviral mechanism(s) of tubacin, the mode of inhibitory action by tubacin was examined using attachment inhibition and time-of-addition assays (Figure 4 and Figure 5; Figures S2 and S3). In attachment inhibition assays, the TE671 cell monolayer was pre-incubated at 4 °C for 10 min, and then reacted with JEV SRIPs (50 TCID50) or virions (50 pfu) plus tubacin (0, 0.1, 5, 10, and 20 μM) at 4 °C for allowing attachment alone. After one hour of incubation, cell monolayer was washed with PBS; residual infectivity of SRIPs and virions was determined using immunofluorescence microscopy and plaque assay, respectively. Real-time fluorescence imaging of SRIP-infected cells indicated that the green fluorescence intensity of SRIP-driven EGFP reporter was very similar between tubacin-treated and mock-treated groups (Figure 4). In addition, the plaque assay for residual infectivity of JEV virions indicated that tubacin had no significant inhibitory effect on residual infectivity compared to controls in the attachment assay (Figure S2). The result of viral attachment assay indicated tubacin did not directly interfere on JEV attachment at early stage of viral replication.

Antiviral mechanism(s) of tubacin against JEV was further evaluated using time-of-addition assays with JEV SRIPs and virions, including (1) pre-treatment (one hour prior to infection), (2) simultaneous treatment (at the same time as infection), and (3) post treatment (one hour post infection) (Figure 5 and Figure S3). The greatest degree of antiviral activity was observed in the mode of pre-treatment with tubacin compared to simultaneous- and post-treatment modes. According to the green fluorescence intensity of SRIP-driven EGFP reporter, IC50 value of tubacin was 1.89 μM in a pre-treatment assay, 4.88 μM in a simultaneous-treatment test, and 2.05 μM in a post-treatment experiment, respectively (Figure 5). Interestingly, post-treatment with tubacin was also very effective in inhibiting the late stage of JEV replication. Therefore, the results indicated that tubacin exhibited preventive and therapeutic activities against JEV infection, implying tubacin as a host-targeting agent to affect the involvement of cellular factors in JEV replication.

### 2.3. Tubacin-Induced Hsp90 Hyperacetylation Was Associated with the Reduction of NS5 RNA Polymerase Activity

**Figure 6:**
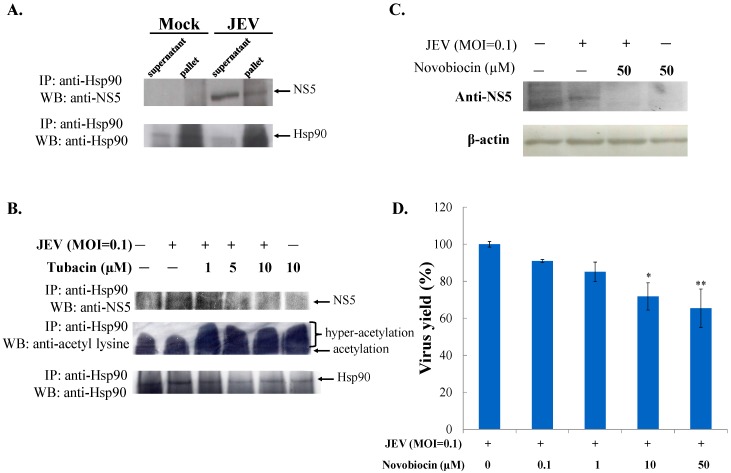
Protein-protein interaction, acetylation level, and function analysis of Hsp90 in JEV replication. For examining the interaction of Hsp90 with JEV NS5, co-immunoprecipitation assay was performed on the infected lysates. After centrifugation, lysate supernatants and precipitated pallets were further analyzed by Western blotting with anti-NS5 and anti-Hsp90 antibodies (**A**); For verifying the acetylation level of Hsp90, JEV infected cells treated with or without tubacin were harvested 36 h post infection; the lysate was accomplished using immunoprecipitation assay with anti-Hsp90 antibodies and protein A-Sepharose beads. The precipitates were examined by Western blotting with anti-NS5, anti-acetyled lysine, and anti-Hsp90 antibodies (**B**); For evaluating JEV NS5 expression in response to novobiocin, Western blotting analysis of infected cells was probed with anti-NS5 antibodies 36 h post infection (**C**); For studying antiviral activity of novobiocin, virus yield in the supernatant of treated infected cells was measured using plaque assays (**D**). * *p* value < 0.05; ** *p* value < 0.01 compared with untreated infected cells.

**Figure 7:**
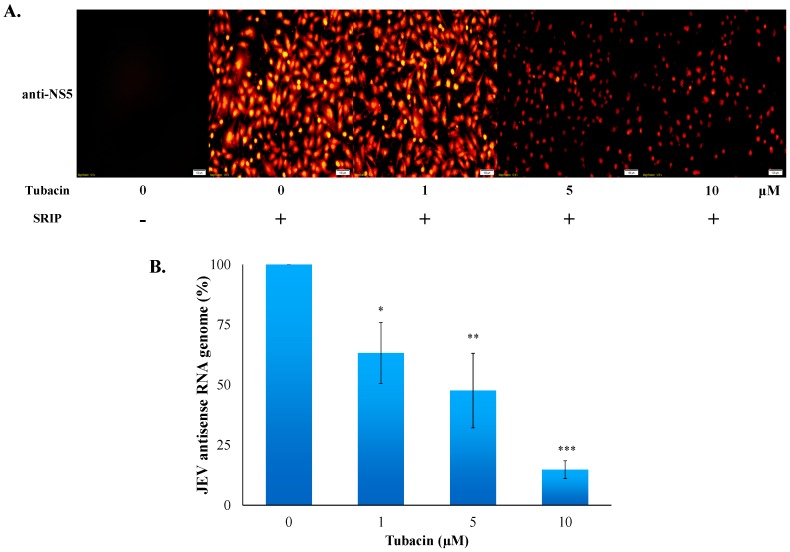
Inhibitory effect of tubacin on the expression of NS5 protein and viral antisense RNA genome in a JEV infected cell. For exploring the NS5 level, infected cells treated with or without tubacin were washed and fixed 36 h post infection, then followed by immunofluorescent staining with anti-NS5 antibodies and Alexa Fluor 546-conjugated secondary antibodies (**A**); For quantitating antisense RNA genomes, total RNAs from the infected cells in the presence and absence of tubacin were performed using real time RT-PCR assay (**B**). * *p* value < 0.05; ** *p* value < 0.01; *** *p* value < 0.001 compared with untreated infected cells. Scale bar = 100 µm.

HDAC6, a cytoplasmic deacetylase, deacetylates several substrates, such as tubulin, Hsp90, β-catenin, and cortactin [24]. Since Hsp90 is suggested as the key chaperone universally required for the homeostasis of viral replication complexes, particular viral RNA-dependent RNA polymerase [25], the protein-protein interaction between host Hsp90 and JEV NS5 RNA polymerase was analyzed with co-immunoprecipitation assays (Figure 6A). Western blotting analysis of co-immunoprecipitates with anti-Hsp90 antibodies demonstrated that JEV NS5 protein in infected cell lysate was co-immunoprecipitated by anti-Hsp90 antibodies (Figure 6A, lane 4 vs. lane 2), revealing that the Hsp90-NS5 interaction might be involved in the JEV replication complex. Hsp90 hyperacetylation via inactivation of HDAC6 causes the loss of chaperone for its binding with Hsp90 client proteins [26,27]. Thus, the interaction of Hsp90 with JEV NS5 as well as Hsp90 acetylation levels in mock and infected cells treated with or without tubacin were also examined using immunoprecipitation assays (Figure 6B). Western blotting analysis indicated that Hsp90 was immunoprecipitated in all samples, and Hsp90 hyperacetylation, with various amounts of acetyl group, was detected in tubacin-treated cells (Figure 6B, lanes 3–6), but mock and infected cells (Figure 6B, lanes 1 and 2). By contrast, the quantity of un-acetylated hsp90 protein decreased post tubacin-treatment (Figure 6B, lanes 3–6). Importantly, the binding ability of Hsp90 to JEV NS5 concentration-dependently decreased post tubacin treatment (Figure 6B, lanes 2–4). Novobiocin, an Hsp90 inhibitor, was used to evaluate the role of Hsp90 in the stability of JEV NS5 protein and virus yield (Figure 6C,D). Novobiocin treatment caused a marked decrease in the amounts of NS5 protein in infected cells (Figure 6C, lane 3 vs. lane 2), as well as reduced JEV yields in a concentration dependent manner (Figure 6D). The results indicated that tubacin induced Hsp90 hyperacetylation and reduced the binding activity of Hsp90 to JEV NS5 in infected cells. The Hsp90 inhibitor, novobiocin, affected the amount of NS5 protein and the JEV production in infected cells. The finding implied that tubacin-induced Hsp90 hyperacetylation might alter the binding interaction of Hsp90 with JEV NS5 which is involved in JEV replication. Subsequently, the protein amount of NS5 and the synthesis of antisense RNA genome in JEV infected cells were determined 36 h post treatment with tubacin using immunofluorescent staining and real-time RT-PCR, respectively (Figure 7). Immunofluorescent staining with anti-JEV NS5 antibodies indicated that tubacin at 5 and 10 µM significantly decreased the protein amount of NS5 in JEV-infected cells (Figure 7A). Meantime, tubacin concentration-dependently reduced NS5-mediated synthesis of JEV antisense RNA genomes in infected cells (Figure 7B). The overall results illustrated that tubacin significantly repressed the functional activity of JEV NS5 in vitro replication.

## 3. Discussion

Selective HDAC6 inhibitors (tubacin and TBSA), but not pan-HDAC inhibitors, exhibited potent antiviral efficacy against JEV in this study. Tubacin and TBSA substantially reduced JEV-induced cytopathic effect and apoptosis, and concentration-dependently lessened virus yields in human cerebellar medulloblastoma cells (Figure 1, Figure 2 and Figure 3). The pan-HDAC inhibitors, such as VPA and arginine butyrate, effectively enhanced the sensitivity of Epstein-Barr virus (EBV)-positive lymphoma cells to ganciclovir [28]. In addition, treatment with pan-HDAC inhibitors (TSA and SAHA) decreased the replication of respiratory syncytial virus (RSV) in vivo [29]. Tubacin, HDAC6-specific inhibitor, dose-dependently boosted the release of influenza A virus (IAV) progeny through the increase of acetylated microtubules for the effective movement of viral components to the plasma membrane [30]. TBSA, a selective HDAC6 inhibitor, caused the acetylated α-tubulin accumulation in hepatocyte cells which was associated with the decrease of hepatitis C virus (HCV) RNA genome synthesis in hepatocyte cells [22]. Therefore, HDACs might be involved in the replication process of many viruses, such EBV, IAV, RSV, and HCV. Moreover, virus-specific HDACs could be the targets for development of virus-distinctive antiviral agents. The study proposed HDAC6 as a JEV-specific host target for exploiting antiviral agents.

Both tubacin and TBSA were selective HDAC6 inhibitors; however, tubacin (IC50 of 0.26 µM in virus yield reduction) was a more potent inhibitor of JEV than TBSA (IC50 of 1.75 µM) (Figure 3). Importantly, tubacin, but not TBSA, markedly reduced the intracellular production of JEV infectious particles in human cerebellar medulloblastoma cells (Figure 3C vs. 3D). The result might be linked with higher HDAC6 selectivity and inhibition potency of tubacin compared to TBSA [31]. Therefore, antiviral activity of tubacin relies on its ability to target a host protein. In a time-of-addition assay, fluorescence intensity of JEV SRIP-driven EGFP reporter and the number of JEV plaques clarified that tubacin pre-treatment showed the highest anti-JEV activity compared to simultaneous treatment and post-treatment (Figure 5, Figure S3). Notably, tubacin post-treatment also significantly suppressed the JEV replication in concentration-dependent manners. The results explicated that a short-term treatment of tubacin presented the potential of the host-targeting agent with preventive and therapeutic activities against JEV infection. Tubacin might be combined with direct-acting antivirals for preventing and treating JE which accomplishes complementary antiviral actions in a synergistic manner and reduces the possibility of viral resistance during clinical therapy.

Hsp90 hyperacetylation induced by an inhibitor of HDAC6/8 MC1568 has been reported to cause the decrease of IAV replication in lung epithelial cells [32]. In this study, JEV NS5 protein was identified as one of the Hsp90 client proteins (Figure 6A). Co-immunoprecipitation indicated that tubacin-treatment caused Hsp90 hyperacetylation and decreased the binding ability of Hsp90 to JEV NS5 in infected cells in concentration-dependent manners (Figure 6B). Tubacin-induced Hsp90 hyperacetylation has been reported to induce the loss of chaperone activity of Hsp90 that resulted in the functional deficiency of Hsp90 client protein glucocorticoid receptor, such ligand binding and transcriptional activation [27]. Hsp90 inhibitor, novobiocin, decreased the protein amounts of NS5 and the virus production in JEV-infected cells (Figure 6C,D). The results indicated the importance of Hsp90-NS5 interaction for JEV NS5-mediated replication. In addition, tubacin treatment caused the diminution of NS5 protein and antisense RNA genome expression in infected cells (Figure 7). Therefore, our results suggested that tubacin-induced Hsp90 hyperacetylation might influence the NS5 activity in JEV replication, as one of the antiviral mechanisms of tubacin against JEV. Hsp90 has also been demonstrated to interact with viral non-structural (except RNA polymerase) and structural proteins to improve the structural folding and functional activity of those viral proteins [25]. Tubacin-induced Hsp90 hyperacetylation could affect the interaction of Hsp90 with JEV proteins, except NS5, involved in RNA translation and replication complex. Interestingly, microtubule acetylation modulated by HDAC6 inhibitors affected the viral replication through interrupting viral components transportation along microtubules [33]. Therefore, the involvement of tubacin-induced hyperacetylation of the other HDAC6 substrates that assist viral translation and replication could not be excluded in the antiviral mechanism(s) of tubacin against JEV.

In conclusion, HDAC6-selective inhibitors exhibited the potential of antiviral activity against JEV. Particularly, tubacin presented the high-potent inhibition of JEV yield (IC50 of 0.26 µM) and intracellular infectious virion production (IC50 of 1.52 µM). Tubacin was demonstrated as a host-targeting agent with preventive and therapeutic activities against JEV. Tubacin treatment caused the decrease of the Hsp90-NS5 interaction and the reduction of viral proteins and antisense RNA genomes in infected cells. The combination of tubacin and direct-acting antiviral agents provides a novel approach for prophylaxis and treatment against JEV infection.

## 4. Methods and Materials

### 4.1. Cells and Virus

Human cerebellar medulloblastoma TE671 cells (kindly provided by Wen-Kuang Yang, China Medical University, Taiwan) used this study were cultured in minimum essential medium (MEM, GE Healthcare Life Sciences, Pittsburgh, PA, USA), 100 U/mL penicillin-streptomycin, and 5% fetal bovine serum (FBS) (ThermoFisher, Waltham, MA, USA). TE671-CprME stable cell line generated in our laboratory is a packing cell line expressing JEV structure proteins (C, prM, and E) which was described in our previous work [34]. TE671-CprME cells were culture in MEM, 5% FBS, and 500 µg/mL G418 (Sigma, Saint Louis, MO, USA). Baby hamster kidney BHK-21 cells for JEV amplification and plaque assay were also grown in in MEM, 5% FBS and 100 U/mL penicillin-streptomycin. All cells were incubated at 37 °C in an atmosphere containing 5% carbon dioxide.

### 4.2. MTT Cytotoxicity Test

HDAC inhibitors TSA, VPA, and TBSA were purchased from Sigma-Aldrich Company; tubacin was obtained from Cayman Chemical Company. Cytotoxicity of HDACi to TE671 and BHK-21 cells was evaluated by MTT (3-(4,5-dimethylthiazol-2-yl)-2,5-diphenyltetrazolium bromide) assay. 5 × 10^4^ cells per well were seeded in 96-well plates and then treated with the indicated concentration of each HDACi. After 48-h of treatment, 25 μL of MTT solution (5 mg/mL) was added to each well and incubated at 37 °C with 5% CO_2_ for 3 h. After three washings with phosphate buffer saline (PBS), 100 μL DMSO was added into each well for dissolving formazan crystals. OD_570−630_ was measured by micro-ELISA reader and survival rate were calculated to indicate suppressive effects of each HDACi on the survival of TE671 and BHK-21 cells. Survival rate (%) = ((Acontrol − Aexperiment)/Acontrol) × 100%. 50% cytotoxic concentration (CC50) values were calculated by computer program (provided by John Spouge, NCBI, NIH).

### 4.3. Inhibitory Assays of HDACi on JEV-Induced Cytopathic Effect and Apoptosis

TE671 cells cultured in 6-well plates were infected with JEV at a multiplicity of infection (MOI) of 0.1 and were treated simultaneously with VPA (1, 1000 μM), TSA (1, 50 μM), tubacin (1, 10 μM), and TBSA (1, 10 μM), respectively. After 36-h of incubation, images of cytopathic effect in each condition were photographed using a microscope. In addition, cells in each well were collected, fixed using 70% ethanol at 4 °C overnight, and then re-suspended in PBS containing 50 μg/mL PI, 0.1 mg/mL RNase and 0.1% Triton X-100. After 30-min of incubation at 37 °C in a darkroom, cell apoptosis (sub-G1 phase) was measured by flow cytometry (Becton-Dickinson, San Jose, CA, USA) at excitation/emission wavelength of 493/636 nm.

### 4.4. Quantitative Assays of Virus Yield and Intracellular Viral Titer

For determining anti-JEV activity of TBSA and tubacin on virus yield, JEV yield in a cultured supernatant of infected cells at an MOI of 0.1 in the presence or absence of TBSA and tubacin was measured using a plaque assay. Serial dilution of the supernatant was added onto BHK-21 cell monolayer in 6-well plates, incubated at 37 °C in 5% CO_2_ for 1 h, and then overlaid with 2 mL MEM medium containing 1.1% methylcellulose. After a 3-day incubation, the cell monolayer was stained with naphthol blue-black dye; in which viral yields were calculated by the number of viral plaques per mL. For measuring the inhibitory effect of TBSA and tubacin on intracellular viral titer, infected cells were harvested after a 36 h treatment with TBSA and tubacin (0, 1, 5, and 10 μM), and then lysed through three freeze-thaw cycles. The intracellular titer of infectious viruses in lysate was measured by the plaque assay mentioned above.

### 4.5. Virus Attachment Assays with Single Run Infectious Particles (SRIPs) and Virions

JEV SRIPs were produced from CprME-expressing packaging cells transfected with JEV replicon containing EGFP reporter [34]. In brief, TE671 cells grown to 90% confluence were transfected with pFlag-CMV3-CprME using Lipofectamine LTX (Invitrogen, Carlsbad, CA, USA); a stable transfected cell line was established after a 10-day-selection with 500 µg/mL of G418. Next, the packaging cells were transfected with pBR322-JEV-EGFP replicon; JEV-EGFP SRIPs were released in cultured media of transfected packaging cells. For the attachment assay, SRIP was mixed with or without tubacin (10 μM), then immediately added to TE671 cell monolayer at 4 °C to allow viral attachment. After 1-h incubation, cells were washed with cold PBS, then incubated with MEM containing 2% FBS at 37 °C in 5% CO_2_. The green fluorescence of the EGFP reporter derived from the replication of JEV SRIPs was detected by fluorescent microscopy at different time courses (0, 6, 12, 24, 30, and 36 h). In addition, the attachment assay was performed with JEV virions, as described in our prior reports [35]. The TE671 cell monolayer in 6-well plates was incubated with the mixtures of JEV (50 pfu) and tubacin (0, 0.1, 5, 10 and 20 μM) at 4 °C for 1 h, the mixture was then removed, washed with cold PBS, and then overlaid with MEM medium containing 1.1% methylcellulose. After a 3-day incubation at 37 °C in 5% CO_2_, the cell monolayer was stained as described in the plaque assay. Residual plaques were counted; the relative percentage of plaque formation was determined as the ratio of plaque number of each tubacin-treated group to that of mock-treated control.

### 4.6. Time-of-Addition Assay

To examine the inhibitory effect of tubacin by time of addition on JEV replication, pretreatment (1 h prior to JEV infection), simultaneous treatment (at the same time as JEV infection), and post treatment (1 h after JEV entry) experiments were performed. For the pretreatment experiment, the TE671 cells monolayer was pretreated with tubacin (0, 1, 5, and 10 μM) for 1 h, and then infected with 10 TCID50 of JEV SRIPs or 100 p.f.u. of JEV virions at 37 °C in 5% CO_2_. The images of replicon-derived EGFP reporter and virus-induced CPE in JEV SRIP-infected cells were taken by fluorescent and optical microscopies 36 h post infection. The fluorescent intensity of EGFP reporter in SRIP-infected cells was quantified by Image J. In the assays with JEV virions, cell monolayers were overlaid with 2 mL MEM medium containing 1.1% methylcellulose. After 72-h of incubation, viral plaques were counted after staining with naphthol blue-black dye. In the simultaneous treatment experiment, cells were simultaneously treated with tubacin and infected with SRIPs or virions. In the post treatment experiment, tubacin was added into cell monolayers 1 h post infection with SRIPs or virions. The following procedures in both these experiments were performed as the pre-treatment test. The photography of EGFP reporter and CPE in SRIP-infected cells was taken using a microscope; plaque number in virion-infected cells was counted after naphthol blue-black dye staining mentioned above.

### 4.7. Detection of Viral NS5 Expression Using Immnunofluorescence

TE671 cells were infected with JEV at an MOI of 0.1, and simultaneously treated with tubacin (0, 1, 5, and 10 μM). Cells were rinsed once with PBS 36 h post infection, fixed with 4% formaldehyde for 30 min, permeabilized with 0.1% Triton X-100, and blocked with 10% BSA in PBS for 1 h at room temperature. Viral NS5 proteins were detected using rabbit polyclonal anti-JEV-NS5 (GeneTex, Inc., Irvine, CA, USA) and secondary AF546 goat anti-rabbit IgG (ThermoFisher). The image of fluorescent signals in treated infected cells was photographed by fluorescent microscopy.

### 4.8. Quantification of Replicon RNA Expression Using RT-PCR

For examining the inhibitory effect of tubacin on the synthesis of viral antisense RNA genomes, total RNAs of TE671 cells with JEV at an MOI of 0.1 were extracted using PureLink Mini Total RNA Purification Kit (ThermoFisher) 36 h post treatment tubacin, reverse transcripted into cDNA with antisense RNA-specific capture primer (5′-GCAGCAGAAGGAAAGACC GTGAT-3′), and followed by measuring antisense RNA genomes using SYBR Green Master Mix kit with JEV-specific primer pairs (5′-TCCACTTCCTCAACGCAATG-3′ at nucleotide 9724–9743 and 5′-CAGTCGTGCCAGCCATG-3 at nucleotide 9799–9783). The Real-time RT-PCR was performed by 7300 Realtime PCR system (Applied Biosystems, Foster City, CA, USA), and then the corresponding threshold cycle value (*C*_t_) was measured. Relative levels of RNA genomes were normalized by the housekeeping gene *GAPDH*, described in a prior report [26]. 

### 4.9. Co-Immunoprecipitation and Western Blotting Assays

Lysate from mock- and JEV-infected cells was incubated with the anti-Hsp90 antibodies (Cell Signaling, Danvers, MA, USA) in a cool room overnight, followed by addition of protein A-Sepharose beads for an additional 4-h. The immunoprecipitate was collected after centrifugation, and analyzed using Western blotting with the anti-JEV NS5 (GeneTex, Inc.), anti-acetyl lysine (Cell Signaling), or anti-Hsp90 antibodies in a cool room overnight. After 4-h of incubation with horseradish peroxidase-conjugated secondary antibodies, the immune-reactive complexes were detected using enhanced chemiluminescence reaction (Amersham Pharmacia Biotech, Piscataway, NJ, USA).

### 4.10. Statistical Analysis

Each data was shown as mean ± standard deviation (S.D.) of three independent experiments. *p* value in each comparison was determined by Student-*t* test, in which the comparison was recognized as a statistical significance if *p* value was lower than 0.05.

## Supplementary Materials

Supplementary materials can be found at www.mdpi.com/1422-0067/18/5/954/s1.

## References

[1] Umenai T., Krzysko R., Bektimirov T.A., Assaad F.A. (1985). Japanese encephalitis: Current worldwide status. Bull. World Health Organ..

[2] Van den Hurk A.F., Ritchie S.A., Mackenzie J.S. (2009). Ecology and geographical expansion of Japanese encephalitis virus. Annu. Rev. Entomol..

[3] Weaver S.C., Reisen W.K. (2010). Present and future arboviral threats. Antivir. Res..

[4] Misra U.K., Kalita J., Goel D., Mathur A. (2003). Clinical, radiological and neurophysiological spectrum of JEV encephalitis and other non-specific encephalitis during post-monsoon period in India. Neurol. India.

[5] Liu T.H., Liang L.C., Wang C.C., Liu H.C., Chen W.J. (2008). The blood-brain barrier in the cerebrum is the initial site for the Japanese encephalitis virus entering the central nervous system. J. Neurovirol..

[6] Liu J.J., Tsai T.H., Chang T.J., Wong M.L. (2003). Cloning and Sequencing of Complete cDNA of Japanese Encephalitis Virus YL Strain in Taiwan. Virus Genes..

[7] Misra U.K., Kalita J. (2010). Overview: Japanese encephalitis. Prog. Neurobiol..

[8] Bessaud M., Pastorino B.A., Peyrefitte C.N., Rolland D., Grandadam M., Tolou H.J. (2006). Functional characterization of the NS2B/NS3 protease complex from seven viruses belonging to different groups inside the genus Flavivirus. Virus Res..

[9] Park G.S., Morris K.L., Hallett R.G., Bloom M.E., Best S.M. (2007). Identification of residues critical for the interferon antagonist function of Langat virus NS5 reveals a role for the RNA-dependent RNA polymerase domain. J. Virol..

[10] Unni S.K., Růžek D., Chhatbar C., Mishra R., Johri M.K., Singh S.K. (2011). Japanese encephalitis virus: From genome to infectome. Microbes Infect..

[11] Okamoto T., Nishimura Y., Ichimura T., Suzuki K., Miyamura T., Suzuki T., Moriishi K., Matsuura Y. (2006). Hepatitis C virus RNA replication is regulated by FKBP8 and Hsp90. EMBO J..

[12] Yang X.J., Seto E. (2008). Lysine acetylation: codified crosstalk with other posttranslational modifications. Mol. Cell.

[13] Krämer O.H. (2009). HDAC2: A critical factor in health and disease. Trends Pharmacol. Sci..

[14] De Ruijter A.J., van Gennip A.H., Caron H.N. (2003). Histone deacetylases (HDACs): Characterization of the classical HDAC family. Biochem. J..

[15] Yang X.J., Gregoire S. (2005). Class II histone deacetylases: From sequence to function, regulation, and clinical implication. Mol. Cell Biol..

[16] Spange S., Wagner T., Heinzel T., Krämer O.H. (2009). Acetylation of non-histone proteins modulates cellular signalling at multiple levels. Int. J. Biochem. Cell Biol..

[17] Liu H.M., Jiang F., Loo Y.M., Hsu S., Hsiang T.Y., Marcotrigiano J., Gale M. (2016). Regulation of Retinoic Acid Inducible Gene-I (RIG-I) Activation by the Histone Deacetylase 6. EBioMedicine.

[18] Miller T.A., Witter D.J., Belvedere S. (2003). Histone Deacetylase Inhibitors. J. Med. Chem..

[19] Dokmanovic M., Clarke C., Marks P.A. (2007). Histone Deacetylase Inhibitors: Overview and Perspectives. Mol. Cancer Res..

[20] Shapira L., Ralph M., Tomer E., Cohen S., Kobiler O. (2016). Histone Deacetylase Inhibitors Reduce the Number of Herpes Simplex Virus-1 Genomes Initiating Expression in Individual Cells. Front. Microbiol..

[21] Kobayashi Y., Gélinas C., Dougherty J.P. (2017). HDAC Inhibitors Containing a Benzamide Functional Group and a Pyridyl Cap are Preferentially Effective HIV-1 Latency Reversing Agents in Primary Resting CD4+ T Cells. J Gen. Virol..

[22] Kozlov M.V., Kleymenova A.A., Konduktorov K.A., Malikova A.Z., Kochetkov S.N. (2014). Selective inhibitor of histone deacetylase 6 (tubastatin A) suppresses proliferation of hepatitis Cvirus replicon in culture of human hepatocytes. Biochemistry (Mosc.).

[23] Ren J.H., Tao Y., Zhang Z.Z., Chen W.X., Cai X.F., Chen K., Ko B.C., Song C.L., Ran L.K., Li W.Y. (2014). Sirtuin 1 regulates hepatitis B virus transcription and replication by targeting transcription factor AP-1. J. Virol..

[24] Li Y., Shin D., Kwon S.H. (2013). Histone deacetylase 6 plays a role as a distinct regulator of diverse cellular processes. FEBS J..

[25] Geller R., Taguwa S., Frydman J. (2012). Broad action of Hsp90 as a host chaperone required for viral replication. Biochim. Biophys. Acta.

[26] Fiskus W., Ren Y., Mohapatra A., Bali P., Mandawat A., Rao R., Herger B., Yang Y., Atadja P., Wu J. (2007). Hydroxamic acid analogue histone deacetylase inhibitors attenuate estrogen receptor-α levels and transcriptional activity: A result of hyperacetylation and inhibition of chaperone function of heat shock protein 90. Clin. Cancer Res..

[27] Kovacs J.J., Murphy P.J., Gaillard S., Zhao X., Wu J.T., Nicchitta C.V., Yoshida M., Toft D.O., Pratt W.B., Yao T.P. (2005). HDAC6 regulates Hsp90 acetylation and chaperone-dependent activation of glucocorticoid receptor. Mol. Cell.

[28] Ghosh S.K., Perrine S.P., Williams R.M., Faller D.V. (2012). Histone deacetylase inhibitors are potent inducers of gene expression in latent EBV andsensitize lymphoma cells to nucleoside antiviral agents. Blood.

[29] Feng Q., Su Z., Song S., Χu H., Zhang B., Yi L., Tian M., Wang H. (2016). Histone deacetylase inhibitors suppress RSV infection and alleviate virus-induced airway inflammation. Int. J. Mol. Med..

[30] Husain M., Cheung C.Y. (2014). Histone deacetylase 6 inhibits influenza A virus release by down-regulating the trafficking of viral components to the plasma membrane via its substrate, acetylated microtubules. J. Virol..

[31] Butler K.V., Kalin J., Brochier C., Vistoli G., Langley B., Kozikowski A.P. (2010). Rational design and simple chemistry yield a superior, neuroprotective HDAC6 inhibitor, tubastatin A. J. Am. Chem. Soc..

[32] Panella S., Marcocci M.E., Celestino I., Valente S., Zwergel C., Li Puma D.D., Nencioni L., Mai A., Palamara A.T., Simonetti G. (2016). MC1568 inhibits HDAC6/8 activity and influenza A virus replication in lung epithelial cells: Role ofHsp90 acetylation. Future Med. Chem..

[33] Zhang L., Ogden A., Aneja R., Zhou J. (2016). Diverse roles of HDAC6 in viral infection: Implications for antiviral therapy. Pharmacol. Ther..

[34] Lu C.Y., Hour M.-J., Wang C.-Y., Huang S.-H., Mu W.-X., Chang Y.-C., Lin C.-W. (2017). Single-Round Infectious Particle Antiviral Screening Assays for the Japanese Encephalitis Virus. Viruses.

[35] Huang S.H., Lien J.C., Chen C.J., Liu Y.C., Wang C.Y., Ping C.F., Lin Y.F., Huang A.C., Lin C.W. (2016). Antiviral Activity of a Novel Compound CW-33 against Japanese Encephalitis Virus through Inhibiting Intracellular Calcium Overload. Int. J. Mol. Sci..

