# Supplementary material for: Tubacin, an HDAC6 Selective Inhibitor, Reduces the Replication of the Japanese Encephalitis Virus via the Decrease of Viral RNA Synthesis
Source: Int J Mol Sci. 2017 May 1;18(5):954. doi: 10.3390/ijms18050954 (PMC5454867; doi:10.3390/ijms18050954)
Supplement: Supplementary file 1 [file ijms-18-00954-s001.pdf]

Tubacin, an HDAC6 Selective Inhibitor, Reduces the Replication of the Japanese Encephalitis Virus via the Decrease of Viral RNA Synthesis

Chien-Yi Lu, Yi-Chih Chang, Chun-Hung Hua, Chieh Chuang, Su-Hua Huang, Szu-Hao Kung, Mann-Jen Hour and Cheng-Wen Lin

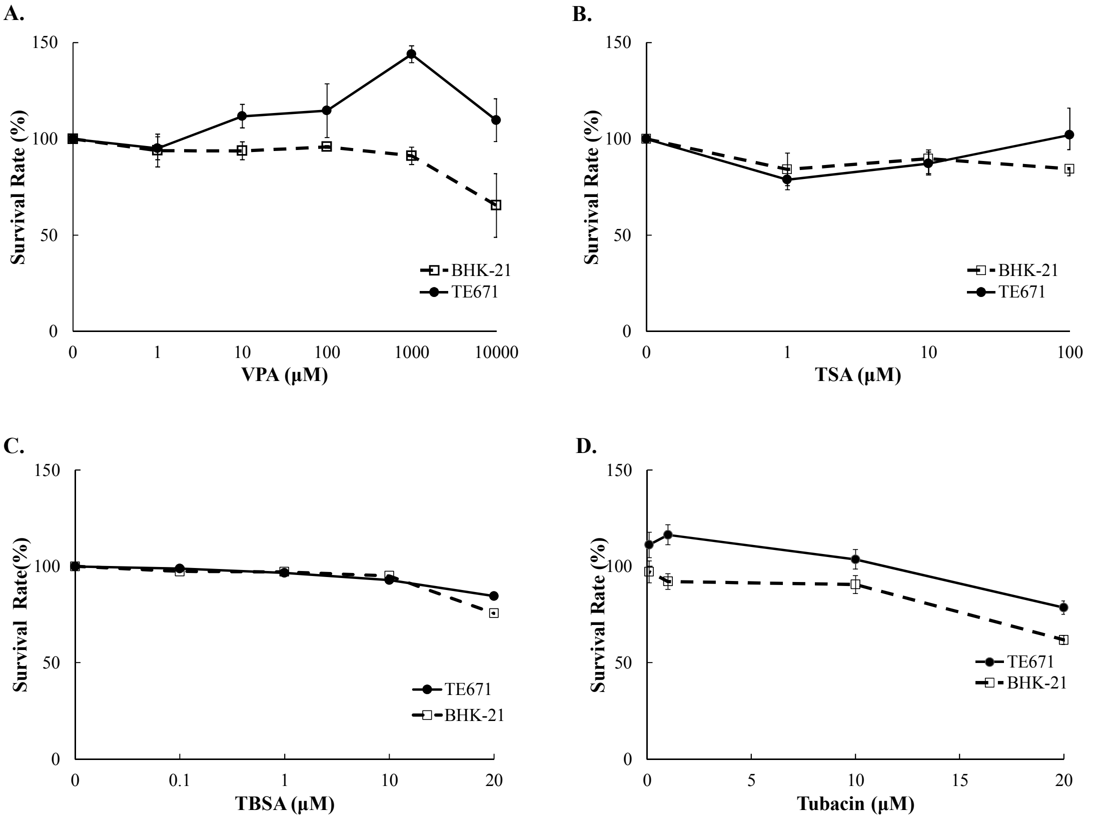

Figure S1. Survival rate of TE671 and BHK-21 cells treated with pan-HDAC and selective HDAC6 inhibitors. Cells were cultured in 96-well plates, treated with VPA (**A**), TSA (**B**), TBSA (**C**), and tubacin (**D**), respectively. After 48 h incubation, the assay was followed by MTT assay. Survival rates of cells were determined as the ratio of $OD_{570-630\ nm}$ of treated cells to $OD_{570-630\ nm}$ of untreated cells.

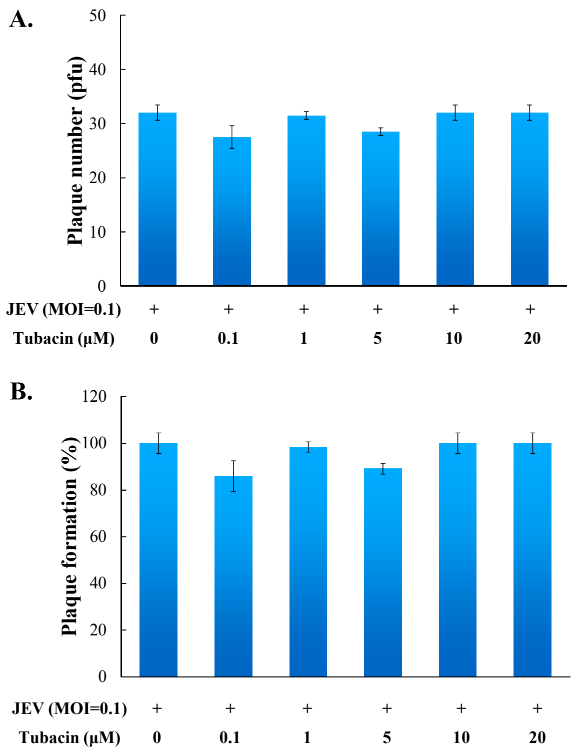

Figure S2. Attachment inhibitory activities of tubacin against JEV. JEV (50 pfu) was mixed with tubacin, and then immediately added onto TE671 cell monolayer. After 1-h incubation at 4 °C, cell monolayer was washed twice with PBS, and then overlaid with 2 mL of a methylcellulose medium for 3 days at 37 °C in CO_2. After staining with naphthol blue-black dye, residual plaques were counted (**A**). Relative percentage of plaque formation was shown based on the ratio of plaque number of each tubacin-treated group to that of mock-treated control (**B**).

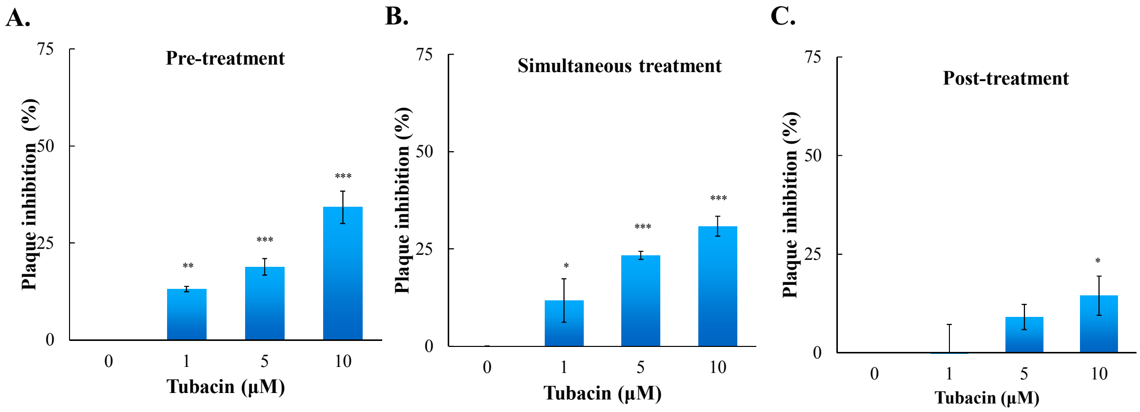

Figure S3. Time-of-addition assays for analysis of antiviral action modes of tubacin against JEV. Infected cells were treated with tubacin 1 h prior (pre) (**A**), simultaneous (**B**), or 1 h post infection (**C**), and then followed by plaque assay. Plaque inhibition was calculated from ratio of treated group to mock-treated control. *, p value < 0.05; **, p value < 0.01; ***, p value < 0.001 compared with untreated cells.
